# Review and Comment on the Relationship between Primo Vascular System and Meridians

**DOI:** 10.1155/2013/279176

**Published:** 2013-05-14

**Authors:** Ding-Jun Cai, Ji Chen, Yi Zhuang, Mai-Lan Liu, Fan-Rong Liang

**Affiliations:** ^1^Chengdu University of Traditional Chinese Medicine, Sichuan, Chengdu 610075, China; ^2^Foreign Languages School, Chengdu University of Traditional Chinese Medicine, Sichuan, Chengdu 610075, China

## Abstract

This paper aims to summarize the recent progress of researches on the primo vascular system (PVS) and to analyze characteristics between PVS and traditional Chinese meridians. With the distribution, position features, identification and origin of PVS, and its function related to meridians elaborated on, we propose that there is still a lack of enough evidence to support the correlation between PVS and traditional Chinese meridians.

## 1. Introduction 

Acupuncture therapy boosts a good clinical efficacy worldwide. According to the traditional Chinese medicine theories, acupuncture owes its favorable effect to the regular and diversified meridians. The meridians are interconnected to form a fundamental system, which are the channels distributing all over the human's body to circulate *qi* and blood and to connect internal viscera with the external part. However, there has emerged little scientific biological evidence to support this traditional theory, since the key problem of meridians seems that what the material basis of the meridians is, which has always been a puzzle for many researchers. The researchers who focus on this problem have labored to expose the structure of meridians in terms of anatomical and physiological methods. 

There were some studies providing few clues to uncover this conundrum. In the early 1960s, Prof. Kim in North Korea proposed that he found the anatomic and physiological basis for the meridians. He stained out some special ducts and nodes in the subcutaneous tissue, organ surface, and nerve tissue, entitled as Bonghan ducts and Bonghan corpuscle, with a novel dye and revealed the morphologic features and function of Bonghan ducts and Bonghan corpuscle in his research [1–6]. However his studies were ignored for almost forty years due to the lack of details in his research protocol, and others failed to reproduce his outcomes. Recently, Prof. Soh's group has carried out a series of studies stemmed from Kim's conjecture of meridians in traditional oriental medicine, which also declared that they had found some structures as primo vascular system (PVS) which was just alike to Prof. Kim's claims. Prof. Soh even pointed out that Bonghan circulatory system serves as an extension of acupuncture meridians [7]. However, as it refers to the relationship between the PVS and meridians, it should be careful due to a few key issues of their distributions, positions, and physiological functions. 

This review is to summarize the recent progress of researches on PVS and to analyze the most important distinguishing characteristics between PVS and traditional meridians. 

## 2. On Universality of PVS Distribution

The PVS is composed of the small primo vessels (PVs) and primo nodes (PNs). It is reported that the PVs are semitranslucent thread-like structures whose diameter is about 0.1 mm connecting the PNs. The PVs away from the PN branch out into 2-3 smaller vessels with fine terminal arborizations [8]. In the past ten years, increasing researches focused on finding out PVS. Various staining techniques, such as Trypan blue techniques [9], Alcian blue dyeing [10], and Janus green B staining method [11] among others, were applied to detect the PVs and PNs, which appeared in most parts of an animal's body. The PVs and PNs were found in nervous system, instance in venous sinuses of rat brains [12], around the perineurium in the spinal cord and in the epineurium, perineurium, and endoneurium of a rat sciatic nerve [9]. They also emerged on the surface of internal organs, for example, stomach, intestine, liver, bladder, and heart [13–17]. In addition, in circulatory system, the PVs and PNs were detected inside the various large blood vessels [18–23] and lymphatic vessels [11, 24–26]. Moreover, the PVs and PNs were observed in the superficial tissues. An example can be found in the hypodermis of rat by using Trypan blue [27]. Besides, beyond the Prof. Kim's hypothesis, in the adipose tissues, PVS was detected [28]. And more interestingly, some researchers reported that the PVs and PNs were not isolated and intermittent, but connected to form a novel network. A significant report stated that the existence of an entire network above the pia mater of the brain and spine of rats was proved with two approaches, spraying Alcian blue into the pia mater of the rat brain and injecting Alcian blue into the lateral ventricle [29]. A similar work also declared that the network of Bonghan ducts (PVs) was noticed in the omentum and peritoneum by using Trypan blue [30]. On the layer of the stratum fibrosum in the superficial fascia of rat hypodermis, the networks of threadlike structures composed of primo nodes and vessels could be found in which the fluorescent nanoparticles existed [31]. The researchers even proposed that the network of PVS in the fascia might be helpful for acupuncture [32]. Those research findings are in accordance with Prof. Kim's conception of the Kyungrak system (meridians) that PVS is an independent functional morphological system in which the superficial PVs and extravascular PVs are connected with superficial nodes, and the deep PVs are connected between them with intravascular PVs, deep PNs, and organ nodes [2]. Based on the above, it seemed that the novel PVS could be considered to have a special relationship with acupuncture meridians due to its distribution features. Unfortunately, a big difference of distribution still lies in PVS and meridians. As it is well known in the oriental traditional medicine, the meridians distribute widely in various parts of the body, and different individuals have a similar distribution of the meridian pathway. Although the above researches have suggested that PVS was observed in most parts of the body, it seems absent in the structures of the organs in head or face. However, according to the classic meridian theory, the six *yang* meridians travel to the head and face and connect with their corresponding body orifices on the head. At the same time, lung and pericardium are important viscera connected with the *yin* meridians. But there has been no reports which stated that the structures of PVS were found in the lung and pericardium. In addition, by carefully analyzing the results of studies that PVS was concerned, it can be found that the PVs and PNs were not observed in all experimental animals, and there even exited gender differences detected in a same experiment [33]. Thus, it seems that there still exists difference in the distribution between the existing research findings on PVS and traditional meridians. Fortunately, some interesting studies may help to answer this question. Primo nodes were observed at CV12, CV10, and CV8, and basic histological study with H&E and Mason's trichrome revealed that they were different from lymph nodes. And after injecting FNP into the primo nodes, it traced the flow of nanoparticles along the CV line to the ligament wrapping the bladder in the primo vessels [34]. After injecting Alcian Blue (AB) dye into the rat acupoint BL23, AB-stained PVS were also observed on the surface of internal organs in right abdominal cavity [35]. In addition, after the subcutaneous injection of fluorescent nanoparticles into the acupoint ST36, the PVs and PNs were found from the knee to the middle of tibia, around the location of the classical “stomach meridian,” of which tracing region was a maximum of 1-2 cm away from the diffusion area [31]. Therefore, despite of the distinct difference of the distribution universality between the PVS and traditional meridians, there is not enough evidence to negate a possible link between these two concepts. In order to discover the relationship between them based on the distribution features, intensive researches should be carried out in future which focus on confirming the existence of PVS in the skin and the ubiquity of the connection between skin PVS, especially acupoints and PVS of internal organs. 

## 3. On Stability of PVS Location

Currently most studies of PVS revealed the existence of PVs and PNs in the animal's body. But the PVs and PNs were floating in liquid. In the blood system, the PVs and PNs were observed floating in the venous sinuses of rats [12], in blood vessel of mice and rats [36], and inside the bovine heart [17]. In the lymph system, the PVs also were floating in the lymphatic vessels. The researchers isolated the floating PVs with diameters of 20~30 *μ*m from abdominal lymph vessels of rabbits by Alcian blue staining [37]. In the nervous system, PVS was not attached to the wall of the ventricle, but acted as a freely floating structure in the cerebrospinal fluid (CSF), and it ran along the central canal of the spinal cord by using fluorescent nanoparticles that were injected into the lateral ventricle [38]. Even on the surface of the internal organs, the PVs and PNs were not adhered to the surface of organs, but were floating in peritoneal fluid [39]. The floating phenomenon indicates that the position of PVS is unfixed and irregular which leads up to irregular observation and hardly reproduction at the same time. However, in accordance with the descriptions of meridians in medical classics, it is not difficult to find that the position of meridians is fixed, stable, and symmetrical under the physiological condition. The irregularly free-floating state of the PVs and PNs was a little bit contradictory to traditional meridian theory. Fortunately, some recent studies dedicated that a fixed PVS of well-defined location was found underneath the superior sagittal sinus in the sagittal fissure of rabbit, and its characteristics were the same as observed in other organs [40]. Moreover, the PVs were irregularly fixed on the stratum fibrosum in the subcutaneous fascia [31]. These findings may be helpful for exploring the fixed PVS in other parts of body and also be beneficial for studying the relationship between PVS and meridians. 

## 4. On Identification and Origin of PVS

Since the novel tread-like structures were observed in the animals, the intensive researches were conducted on the field which distinguished those semitransparent tread-like structures from other tissues. Because, inside an organism, there are many similar tread-like structures, especially under certain special experimental conditions. In both Prof. Kim' and Prof. Soh' researches, the method of blood perfusion was adapted to study PVS in the blood vessels. Injecting a 10% dextrose solution into the left femoral vein to replace blood, the retaining PVs in the vessels were observed which were longitudinal tread-like structures floating in the transparent fluid [18–20]. Some researchers also found some similar fibrous string in the vessels through blood perfusion by giving 0.9% NaCl solution into femoral vein of rabbit on one side. However, their further studies suggested that the fibrous strings were coagulated composed by bundle of fibrin because of distinct difference of tracing rate between injecting hypercoagulable and hypocoagulable perfusion fluid [41]. Meanwhile, with careful analysis on the method of Soh's research team, some researchers found that PVS on a visceral organ surface was frequently observed in phenylhydrazine- (PHZ-) induced anemic rats [7]. But phenylhydrazine is known to cause hypercoagulability. They designed to investigate PVS on PHZ-induced anemic and bleeding-induced anemic rat models. The tread-like structures were only detected on PHZ-induced anemic and the phenomena were blocked when heparin was administered [42]. It is well known that even slightly bleeding could make it easy to find tread-like structures on the internal organs. Since it is badly difficult in avoiding bleeding during surgical operation, it is crucial to identify PVS from coagulate string. Concerned with Prof. Kim's hypothesis, Prof. Soh's research group confirmed PVS by some distinctive characteristics, such as tread-like structures connected with node, multiple tubular structure, trypan blue sustainability, and rod-shaped nuclei. But the rod-shaped nuclei are absent in fibrin [43]. Conversely, research results stated that the tread-like structures detected under hypercoagulability and blocked by heparin also have the four hallmarks of the PVs [42]. 

There is another condition to which the special attention should be attracted. The visceral peritoneum is easily damaged by mechanical and chemical factors during the operation. Therefore, it is necessary to distinguish the internal organ-surface PVS from torn peritoneum and the debris of peritoneum. Using stereoscopic and microscopic observation, some researchers discriminated internal organ-surface primo vessels from torn mesentery, which is considered as a similar and potentially confusing tissue [44]. Both of them are milky-white-colored and string-like observed by a stereoscope. But there are distinguishing features between them. The internal organ-surface PVs were weakly connected to the organ surface, connected to corpuscles, and branched onto the surface of other organs. While torn mesentery tightly attaches to the organ surface with a fan-shape membrane, which is strong enough to withstand force sufficient to lift the organ, and does not branch. Moreover, the PVs were bundle patterns, while irregular patterns were seen in torn mesentery tissue under an optical microscope. To our knowledge, some anesthetics, such as urethane, can lead up to injury of visceral peritoneum by IP injection. In most studies of PVS on organs' surface, animal models were usually anesthetized by administrating anesthetic directly into abdominal cavity. Thereby, the debris of visceral peritoneum should be considered. A research revealed that the debris of visceral peritoneum caused by urethane was thread-like structures in the peritoneal fluid, which also had the above four characteristics of PVS [42]. The research results seemed controversial, as this may be due to the loose criteria of PVS. According to the Kim's conception and traditional meridian theory, PVS and meridians should be continuous and form a certain network, and the tread-like structures in the peritoneal fluid were always fragment, which might be considered to be the segmental structures from the allover of PVS in the peritoneum and omentum. Hence, there is a need to develop more suitable methods to identify PVS from these similar tissues. 

Furthermore, one more easily confusing tissue is the lymph vessel. Both lymph vessels and the PVs are transparent vessels. But there are many characteristics of the PVs different from the lymphatic vessels [26, 37, 45, 46]. The first and most importance is that the PV has a multiple tubular structure and is filled with fibrous material, while the lymph vessel is a single tube. Secondly, the PV reveals rod-shaped nuclei stained by Acridine orange and 4′,6-diamidino-2-phenylindole and dihydrochloride (DAPI). And the blue-stained nuclei, which are distributed in a broken-lined stripe, form a tube structure. Thirdly, the size of them is different. For example, the average diameter of the lymph vessels inside the caudal vena cava of the rabbits was 258.5 *μ*m and the average diameter of the primo vessels was 26 *μ*m. Fourthly, the PV is easy to lift from internal organ surface, while the lymphatic vessel is fixed in the organ. Fifthly, the PV has some sinuses through which some liquid and granules flow. Last but not the least, by applying immunostaining with a lymphatic marker of lymphatics, LYVE-1, the lymphatic endothelial cells with strong positive staining are clearly located at the inner boundary of the lymphatic vessel whereas no LYVE-1 positive cells were detected in the PV. In addition, compared with the blood vessel, the PV has distinguished features which are multilumen structure from the rod-shaped nuclei of endothelial cells [46]. All of these research findings revealed that PVS was a novel and independence structure in the animal's body although some uncertain conflict still existed. 

Moreover, a question concerned is what the origin of PVS is. However, the current research results seemed to be contradictory. Using double-labeled, positive green fluorescence and Trypan blue staining, a research finding stated that PVS in the induced tumor in a green fluorescence protein- (GFP-) expressing mouse originated from endogenous sources rather than from exogenous cells, because PVS shared the genetic characteristics of the host animal. At the same time, with the fluorescence microscopy employed to investigate the PVs in the epidermis under the skin in the normal GFP mouse, an extensive length of the PV line and a PN was clearly observed, which was surrounded with abundant adipose tissue. Combined the finding in the normal GFP mouse, it would be more certain that PVS is endogenous [47]. On the contrary, a research in 2013 suggested a different view. Through immunostaining with antibodies against human CD3 (T lymphocyte), CD20 (B lymphocyte), CD45 (histiocyte), CD68 (macrophage), and lysozyme, it was found that the cells in the PNs of the mice with human U937 tumor were strongly immunoreactive to lysozyme and modestly immunopositive for CD45 and CD68. The author pointed out that the high expression of lysozyme, which was specific for histiocytic lymphoma, in PVS cells strongly suggested that PVS was not of host origin but derived from the xenografted tumor cells. Meanwhile, qRT-PCR analysis of mRNA isolated from PVS cells also revealed a striking predominance of human, rather than mouse, sequences [48]. According to Kim's hypothesis, PVS would vary in different conditions, which is in accordance with the classic meridian theory. But this change does not alter the feature that the meridians or PVS belong to an inherent structure of body. Given that PVS is a basis of traditional meridians, it should be an intrinsic structure in any states. It seemed that the last research did not support the above opinion. However, combined with these two researches, the results benefited us to understand the origins of the different components of PVS. The primo vessels originated from endogenous sources. The cells in the primo nodes might change in different conditions, just like the physical and chemical features of the acupoints would vary under pathological condition [49]. And more interestingly, the finding of the research focused on the markers of the epithelium and endothelium is that the PN and PV (BHC/D) from within lymphatics and those on organ surfaces have the same wall structure, suggesting that they have the same developmental origin [50]. This is also valuable for regarding PVS all over the body as a whole like meridians. 

## 5. On Function of PVS Related to Meridian 

A series of PVS studies suggested that PVS has certain physiological functions. According to Prof. Kim [2–5], the functions of PVS (Kyungrak system) included many aspects, for example, (1) the circulatory function of PVS; (2) PVs with bioelectrical activity, excitatory conductivity, and mechanical motility; (3) hematopoietic function for the intravascular PVS; (4) the biochemical function of the primo fluid; (5) the regeneration of damaged function of primo microcells (Sanals). The researcher also has summarized the functions of PVS in his article, including carrying a fluid, immune action, a potential effect of cells, and development and differentiation of organs [51]. In traditional meridian theory, the significant function of meridians is to convey *qi* and blood and to receive stimulus to regulate the functions of organism. Therefore, the function of PVS which might be associated with these two aspects will be discussed further. 

Based on the morphological studies, it is confirmed that the PVs are bundle pattern of tubules filled with primo fluid [16, 52–54]. By injecting fluorescent nanoparticles into a PN (BH corpuscle), the experiment showed a one-way flow, but the flow speed was not measured properly because of the limitation of experimental conditions [55]. Another research measured the flow speed by injecting Alcian blue into the PN (BHC) on the surface of rabbit's liver. The flow was unidirectional whose distance was up to 12 cm, and the speed was measured as 0.3 ± 0.1 mm/s [16]. The results were consistent with the Prof. Kim' work. According to traditional theory of meridians, the meridians perform the function to carry *qi* and blood to organs and tissues. And the research on propagated sensation along meridians also revealed the transmission with low-speed characteristics [56]. Therefore, from this perspective, PVS and meridians are considered to link with each other potentially. 

The principal function of meridians and acupoints is to receive stimulation and induce therapeutic effect, especially after receiving appropriate stimulations. Provided the established connection between the external stimulation and internal organ's effect, PVS might be like a foundation of meridians. Fortunately, a few researchers have devoted themselves to solving this issue. A research team focused on the electrophysiological characteristics of PVS. Applying extracellular recording method, the researchers recorded two types of pulses generated by the primo vessels on the internal organ surface: type I pulses' feature was with fast depolarizing and repolarizing phases, and type II pulses' characteristic was with fast depolarizing phase and gradually slowing repolarizing phase. And moreover, basing on the sharp top and larger amplitude of the pulses induced by stimulating primo vessels, the researchers could distinguish them from the pulses generated by smooth muscle [57]. Using intracellular recording method, others found that the electrical potential rose slowly by an average of 10.5 ± 8.4 mV in 18.1 ± 14.0 seconds to a steady resting potential, and irregular bursts of spontaneously evoked spikes occurred in the resting potential with an average duration of 16.6 ± 14.9 seconds. The average amplitude of the spikes was 1.2 ± 0.6 mV, and the average duration of the spikes was 0.8 ± 0.8 seconds while the full width at half height was 0.27 ± 0.19 seconds. These results implied that the resting potential of a PN not only was merely to be smooth-muscle-like, but also acted as the irregular burst pattern of nerve tissue [58]. A morphological work which found the presence of nerve-like structures confined to the PNs also supported the above result [48]. Further research suggested that there are different types of cells in PVs and PNs and some of them were excitable [59]. All of these researches suggested that PVS was excitable, which had capability of receiving stimulation and generating a certain reaction. This function might benefit information transfer induced by external stimuli through PVS. 

However, whether this kind of electrophysiological reaction generated by the PVs on the internal organ surface could be induced by subcutaneous PV and PN or acupoints is still uncertain. This point is highly important for acupuncture treatment. Hence, another research focused on the relation between the PVs on the internal organ surface and acupoints through detecting the modulation of gastric motility by stimulating the PVs on the surface of stomach or intestine, as well as acupoints *Zusanli* (ST36) and *Zhongwan* (CV12). The researchers based the view that the PVs on the surface of stomach or intestine did not mediate the regulation of gastric motility induced by stimulating at the acupoints ST36 or CV12 on the fact that electric stimulation of the PVs had no effect on the gastric motility and on the fact that the effect of stimulating at CV12 or at ST36 is no significant difference between the PVS-intact and the PVS-cut rats [60]. Combined with the above research works, it is conceived that lacking the function of the PVs induced by stimulating acupoints makes it fail to support the relationship between PVS and meridians even though PVS could be excitable. However, there are some researches contributing a different view. They suggested that the PVs and PNs on the surface of organs formed a closed circulatory system, within which there were many kinds of immune cells, such as mast cells (20%), eosinophils (16%), neutrophils (5%), and histiocytes (53%) [50]. Therefore, they proposed that the main function of PVS on the organs' surfaces was to regulate immune function, which was in accordance with Prof. Kim's claim. But based on Prof. Kim's conception, all the nuclei of tissue cells are connected with fine terminal subducts and these subducts are connected to the primo vessels for the organs. Acupuncture may regulate organs' function by simulating exterior PVs and PNs through the exterior tissue cells. The proteomic analysis of the PVs also showed that the PVs had some protein whose duty was responding to stimulus [61]. Furthermore, the histological research of PV and PN also showed that there was abundant fibrillar material composed of thread-like structures suggestive of collagen and/or elastic fibers. It is known that the connective tissue is the carrier of the mechanical stimulation induced by acupuncture [62, 63]. Hence, further researches on acupuncture regulating organs' functions through the exterior-interior PVS should be conducted in future. Only by establishing the functional connection of the exterior-interior PVS between the stimulus of acupoints and responds of organs could PVS be a basis for meridians.

## 6. Conclusions 

 We reviewed the features of PVS in light of its distribution, which suggested that PVS covered the massive parts of the body. However, the relevant skin PVS has not been studied completely, and only few researches revealed some cues. Thus, a comparison between acupuncture meridians and PVS leaves nothing rigorous but a mist. BH Kim's claim on the observation of acupuncture meridians remains to verified in future. We analyze the position stability, identification, and origin of PVS. The data demonstrate that PVS is a novel and distinctive structure, but the criteria of it are still needed to develop. The locations of the PVS subsystems floating in fluid are not fixed, and those fixed-location PVS subsystems like intraorgan PVS are not yet observed. The origin of the primo vessels and nodes associated with xenografted tumor is the host animal, but cells like the histiocytes in the primo node are from the tumor. We pay more attention to the function of PVS related to meridians. The study with PVS on the organ surface showed that they are not involved with acupuncture stimulations, and further studies with skin PVS and extra PVS are required to find out the functional relation with acupuncture. In conclusion, there is still a lack of enough evidence to ensure PVS as a fundamental substance of traditional meridians. In order to verify the hypothesis of the relationship between PVS and meridians, further researches on the skin PVS, extra PVS, and the functional connection between them and organs' function should be carried out in the near future. 
